# Activation of the Mammalian Target of Rapamycin in the Rostral Ventromedial Medulla Contributes to the Maintenance of Nerve Injury-Induced Neuropathic Pain in Rat

**DOI:** 10.1155/2015/394820

**Published:** 2015-12-06

**Authors:** Jian Wang, Da-Yun Feng, Zhi-Hua Li, Ban Feng, Han Zhang, Ting Zhang, Tao Chen, Yun-Qing Li

**Affiliations:** ^1^Department of Anatomy and K. K. Leung Brain Research Centre, Fourth Military Medical University, Xi'an 710000, China; ^2^Department of Neurosurgery, Tangdu Hospital, Fourth Military Medical University, Xi'an 710000, China; ^3^Basic Medical College, Zhengzhou University, Zhengzhou 450001, China; ^4^Collaborative Innovation Center for Brain Science, Fudan University, Shanghai 200032, China

## Abstract

The mammalian target of rapamycin (mTOR), a serine-threonine protein kinase, integrates extracellular signals, thereby modulating several physiological and pathological processes, including pain. Previous studies have suggested that rapamycin (an mTOR inhibitor) can attenuate nociceptive behaviors in many pain models, most likely at the spinal cord level. However, the mechanisms of mTOR at the supraspinal level, particularly at the level of the rostral ventromedial medulla (RVM), remain unclear. Thus, the aim of this study was to elucidate the role of mTOR in the RVM, a key relay region for the descending pain control pathway, under neuropathic pain conditions. Phosphorylated mTOR was mainly expressed in serotonergic spinally projecting neurons and was significantly increased in the RVM after spared nerve injury- (SNI-) induced neuropathic pain. Moreover, in SNI rat brain slices, rapamycin infusion both decreased the amplitude instead of the frequency of spontaneous excitatory postsynaptic currents and reduced the numbers of action potentials in serotonergic neurons. Finally, intra-RVM microinjection of rapamycin effectively alleviated established mechanical allodynia but failed to affect the development of neuropathic pain. In conclusion, our data provide strong evidence for the role of mTOR in the RVM in nerve injury-induced neuropathic pain, indicating a novel mechanism of mTOR inhibitor-induced analgesia.

## 1. Introduction

The rostral ventromedial medulla (RVM) is an important relay region that contributes to the descending pain control pathway from the periaqueductal gray (PAG) to the superficial laminae (laminae I and II) of the spinal cord [1, 2]. It is well known that the RVM is closely linked to long-lasting activation of descending control circuits that involve descending facilitation, which significantly contributes to the development of persistent pain induced by tissue and nerve injury [3]. Although many studies have focused on this region, the cellular and molecular mechanisms of descending pain facilitation control remain poorly understood.

Due to the role of the descending pain facilitation pathway, several types of injuries, such as tissue and nerve injury, often become chronic and persistent [4], eventually leading to neuropathic pain. The neuropathic pain impairs quality of life and imposes high societal costs. To date, significant progress has been made in basic and clinical studies; however, the currently available therapies for neuropathic pain remain inadequate, and the search continues not only for improved treatments but also for novel targets.

The mammalian target of rapamycin (mTOR), a conserved serine-threonine protein kinase that is inhibited by the effective clinical immunosuppressant rapamycin, regulates several intracellular processes in response to various extracellular signals and thereby modulates mRNA translation. Thus, mTOR plays a critical role in the modulation of long-term plasticity and memory processes [5–7]. Activation of the mTOR complex with the protein raptor (mTORC1) promotes the phosphorylation of mTOR downstream targets, including eukaryotic initiation factor 4E-binding protein (4E-BP1/2) and S6 kinase (S6K), which can further lead to local protein synthesis. It has been reported that deletion of either the 4E-BP1/2 or the S6K gene in mice results in deficits in synaptic plasticity and long-term memory [8, 9]. Moreover, phosphorylated mTOR (p-mTOR), which is the activated form, is upregulated in the peripheral nervous system as well as at the spinal cord level in several pain models [10–15]. Inhibition of spinal cord mTOR by intrathecal administration has proven to be effective in alleviating the nociceptive behaviors of animals under pain conditions [10, 11, 16, 17]. Synaptic plasticity changes in chronic pain conditions can occur not only at the spinal cord level but also at the supraspinal level, including the RVM. Therefore, considering the important role of the RVM in descending pain facilitation, targeting mTOR in the RVM might be a promising way to combat pain.

Because serotoninergic (5-HTergic) neurons are the primary constitutive element in the RVM and can send projections to the superficial spinal dorsal horn (SDH) [18–20], we thus hypothesize that 5-HTergic spinally projecting neurons in the RVM contain mTOR, the activation of which could in turn increase the excitability of the 5-HTergic neurons and thus may potently potentiate the descending facilitation pain control pathway and exaggerate neuropathic pain conditions.

Accordingly, we used a spared nerve injury (SNI) model to evaluate the role of mTOR in the RVM in neuropathic pain in rats.

## 2. Materials and Methods

### 2.1. Animals

Adult male Sprague-Dawley (SD) rats (weighing 250–290 g) were used in the present study. The Ethics Committee for Animal Experiments of the Fourth Military Medical University (Xi'an, China) approved the animal experiments (permit number: 10071). All procedures were in agreement with the IASP guidelines [21]. Efforts were made to minimize the number of animals used and their suffering.

### 2.2. Establishment of SNI Model

The SNI surgery was performed as reported previously [22, 23]. Briefly, rats were anesthetized with pentobarbital (45 mg/kg,* i.p.*), and three terminal branches of the sciatic nerve were exposed by direct incision of the skin and a section of the biceps femoris muscle in the left thigh. The tibial and common peroneal branches were carefully tight-ligated with 5-0 silk sutures and sectioned distal to the ligation, removing 2–4 mm of the distal nerve stump. Muscle and skin were closed in two layers. The surgical procedures for the sham-operated group were identical to those for the SNI group, except that the nerves were not lesioned.

### 2.3. Behavioral Tests

Mechanical allodynia, as a behavioral sign of SNI-induced neuropathic pain, was assessed by measuring the 50% paw withdrawal threshold (PWT) as described previously [24]. The 50% PWT in response to a series of ascending von Frey filaments (Stoelting, Kiel, WI, USA) was determined by the up-and-down method [25]. Von Frey force was delivered perpendicularly to the plantar surface of the hind paw for 2 to 3 seconds. An abrupt withdrawal of the hind paw during stimulation was recorded as a positive response. The 50% PWT was calculated using the following formula: 50%  PWT = 10^[*Xf* + *kδ*]^. Mechanical allodynia was assessed by measuring the 50% PWT of the ipsilateral hind paw in SNI- or sham-operated rats.

Thermal hyperalgesia of the hind paws was tested as described in a previous report [26]. A radiant heat source was focused onto the plantar surface of the hind paw. Measurements of the paw withdrawal latency (PWL) were obtained using a timer that was started by the activation of the heat source and stopped when withdrawal of the paw was detected with a photodetector. Three measurements of the PWL were taken for each hind paw and were averaged as the result of each test session. The ipsilateral hind paw was tested with intervals of more than 5 min between consecutive tests.

### 2.4. Neuronal Tract Tracing

Fluoro-gold (FG) was used as a retrograde tracer to label the RVM neurons that project to the SDH. The procedures for FG injection were essentially the same as in our previous studies [20, 27]. Briefly, after exposing the lumbar cord, 0.1 *μ*L of a 4% solution of FG (Fluorochrome, Denver, CO, USA) dissolved in 0.9% saline was stereotaxically injected into the left side of the lumbar dorsal horn with a microsyringe attached to a glass micropipette by pressure injection. Due to the transportation period of the tracer, the rats were allowed to recover and survive for 7 days.

After perfusion, the brains and spinal cords of the rats were cut into sections. The sections were used to evaluate the FG injection sites in the SDH as well as the distribution patterns of the retrogradely FG-labeled neurons in the RVM under an epifluorescence microscope (BX-60; Olympus, Tokyo, Japan) using an appropriate filter for FG (excitation 350–395 nm; emission 430 nm).

### 2.5. Cannula Implantation and Microinjection into the RVM

After the rats were anesthetized with pentobarbital (45 mg/kg,* i.p.*), a 26-gauge stainless steel guide cannula was stereotaxically implanted into a site above the RVM (10.52 mm posterior to bregma, 0 mm lateral from the midline, and 10.20 mm beneath the surface of the skull). The rats were given one week to recover after cannula implantation. Intra-RVM microinjections were delivered via a 33-gauge injector needle cannula that was lowered 0.5 mm deeper into the brainstem than the guide cannula. The microinjection apparatus consisted of a Hamilton syringe (10 *μ*L) connected to an injector (33-gauge) by a thin polyethylene tube and a motorized syringe pump. Rapamycin (250 *μ*M/1 *μ*L, dissolved in a saline/DMSO mix comprising 25% DMSO, Tocris Bioscience, Minneapolis, MN, USA), the specific inhibitor for mTOR, was infused into the RVM at a rate of 0.1 *μ*L/min; an equivalent volume of 25% DMSO was used as a vehicle. After injection, the microinjection needle was left in place for at least 2 min. The injection sites were verified at the end of all of the experiments by Nissl staining, and injection sites outside the RVM region were excluded from the study. The total number of rats showing successful implantation in the target was 28.

These rats were randomly divided into two groups designed for different purposes, as shown in Figure 6(c): Group 1: to investigate whether rapamycin could influence the induction stage of SNI-induced neuropathic pain, behavioral tests were performed before the first drug or vehicle injection, followed by SNI (Pre-SNI), and 30 min after the second injection on day 1 after SNI (SNI-D1) (Figure 6(c), top) and Group 2: behavioral tests were performed before SNI (Pre-SNI), 6 days after SNI (SNI-D6), and 30 min after drug or vehicle injection on day 7 after SNI (SNI-D7) for the purpose of demonstrating the effect of rapamycin on the maintenance stage of SNI-induced neuropathic pain (Figure 6(c), bottom).

### 2.6. Immunofluorescent Histochemical Staining

The rats were transcardially perfused with 150 mL of 0.01 M phosphate-buffered saline (PBS, pH 7.4), followed by 500 mL of 4% paraformaldehyde in 0.1 M phosphate buffer (PB, pH 7.4). The brainstems and/or spinal cords were transversely sliced into 25 mm thick coronal sections using a freezing microtome (CM1950, Leica, Heidelberg, Germany).

Double-immunofluorescence staining for p-mTOR/NeuN, p-mTOR/FG, or p-mTOR/5-HT was performed. All the antisera used here are shown in Table 1. The sections were sequentially incubated at room temperature with primary antisera in 0.01 M PBS containing 5% normal donkey serum (NDS), 0.3% Triton X-100, 0.05% NaN_3_, and 0.25% carrageenan (PBS-NDS, pH 7.4) for 24 h. Then, the sections were incubated with fluorescein-labeled IgG (secondary antisera) for 6 h. A negative control experiment, in which the primary antisera were omitted, and a peptide competition assay were both carried out. No immunopositive products were detected.

After the immunofluorescence histochemical staining, the sections were observed and images were captured using a confocal laser-scanning microscope (CLSM, FV1000, Olympus). Digital images were captured using FLUOVIEW software (Olympus).

Micrographs of 10–12 sections per rat, which were 150 *μ*m apart within bregma −9.30 to −11.60 mm, were analyzed for p-mTOR expression. Using ImageJ software, the RVM area, including the nucleus raphe magnus (RMg) and the nucleus reticularis gigantocellularis pars *α*, was outlined based on the Nissl staining. The p-mTOR-positive cells within the area were counted manually by an observer blinded to the treatment conditions. The same counting method was used to evaluate the coexpression of 5-HT/p-mTOR as well as that of p-mTOR/FG within the RVM. Cells with visible green cytoplasmic staining represent 5-HTergic or FG-labeled cells, while red staining represents p-mTOR-positive cells; thus, cells with 5-HT or FG double-labeling with p-mTOR appear yellow.

### 2.7. Brain Slice Preparation

The rats were decapitated, and brain slices (400 mm) containing the RVM were cut at 0°C with a vibratome (VT1200s, Leica) in a sucrose cutting solution containing the following (in mM): KCl 2.5, NaH_2_PO_4_ 1.2, NaHCO_3_ 26, sucrose 252, MgSO_4_·7H_2_O 6, CaCl_2_ 0.5, and glucose 10, bubbled with 95% O_2_/5% CO_2_ (pH 7.4). For the electrophysiology studies, the brain slices were transferred to a submerged recovery chamber with oxygenated artificial cerebrospinal fluid (ACSF) containing the following (in mM): NaCl 124, KCl 2.5, MgSO_4_·7H_2_O 2, NaH_2_PO_4_ 1, NaHCO_3_ 25, CaCl_2_ 2, and glucose 37 for 2 hours at room temperature before recording. For the biochemical experiments, the slices were slowly brought to a final temperature of 30°C in ACSF gassed with 95% O_2_/5% CO_2_ and incubated for at least 1 hour before the experiments. The brain slices were treated with rapamycin (250 *μ*M) and vehicle for 30 min. Subsequently, the RVM regions were microdissected and snap-frozen over dry ice.

### 2.8. Western Blot Assay

Following the standard western blot protocol, rats were anesthetized with an overdose of pentobarbital (60 mg/kg,* i.p.*), and the RVM regions were carefully dissected and harvested for western blotting. To obtain total protein extracts, the tissues were lysed in 300 *μ*L lysis buffer containing 10 mM Tris, 150 mM NaCl, 1% Triton X-100, 0.5% NP-40, and 1 mM EDTA at pH 7.4. The samples were adequately mixed at a 100 : 1 (v/v) ratio of protease inhibitor cocktail and phosphatase inhibitor cocktail (Roche, Tucson, AZ, USA). The procedures for the* in vitro*-infused brain slices were similar to those of the tissue protocols. The samples were stored at −80°C for western blot analysis. Then, 30 *μ*g of cell lysis material (quantitatively measured using the BCA protein assay; Thermo Scientific, Rockford, IL, USA) was resolved by sodium dodecyl sulfate-polyacrylamide gel electrophoresis (SDS-PAGE) and transferred to PVDF membranes (Immobilon-P, Millipore). After blocking in nonfat milk for 1 h, the membranes were incubated overnight at 4°C with the following primary antibodies: rabbit anti-mTOR (1 : 1000, Cell Signaling Technology); rabbit anti-p-mTOR (1 : 1000, Cell Signaling Technology); rabbit anti-S6K (1 : 1000, Cell Signaling Technology); rabbit anti-p-S6K (1 : 1000, Cell Signaling Technology); and mouse anti-*β*-actin (1 : 5000, Sigma, St. Louis, MO, USA). The immunoblots were then reacted with the corresponding horseradish peroxidase- (HRP-) conjugated secondary antibodies (anti-rabbit 1 : 5000, anti-mouse 1 : 5000; Amersham Pharmacia Biotech, Piscataway, NJ, USA). All of the reactions were detected by the enhanced chemiluminescence (ECL) detection method (Amersham) and exposure to film. The scanned images were quantified and analyzed with ImageJ software. Target protein levels were normalized against *β*-actin levels and expressed as fold changes relative to the naïve control group.

### 2.9. Electrophysiology

Neurons in the RVM region were targeted for recording using an upright microscope equipped with Zeiss (Oberkochen, Germany) infrared-differential interference contrast (IR-DIC) optics, a 40× water-immersion objective, and a video-imaging camera. The patch pipette was filled with intracellular solution containing the following (in mM): K-gluconate 130, NaCl 5, KCl 15, EGTA 0.4, HEPES 10, Mg-ATP 4, and Tris-GTP 0.2, pH 7.25–7.35, with an osmotic pressure of 290–300 mOsm/L. The pipette resistance, as measured in the bath, was typically 4 ± 0.5 MΩ. The voltage was held at −60 mV, and neurons were given at least 3 min to stabilize before data were collected. Spontaneous discharge and the number of action potentials were used to investigate SNI-induced changes in neuronal excitability in the RVM. The initial access resistance was 15–30 MΩ and was monitored throughout the experiment. Data were discarded if the access resistance changed by >15% during the experiment. Data were filtered at 1 kHz and digitized at 10 kHz.

#### 2.9.1. Spontaneous Discharge

The excitatory postsynaptic currents (EPSCs) of the RVM 5-HTergic neurons, which are mediated by AMPA receptors [28], were voltage-clamped and recorded at −60 mV with an Axon 700B amplifier (Molecular Devices, Sunnyvale, CA, USA) after blocking GABAergic transmission by picrotoxin (100 mM, Sigma), a GABA_A_ receptor antagonist.

#### 2.9.2. Spike Number

The membrane excitability of the recorded neurons was measured in current-clamp mode by determining the number of action potentials elicited by intracellular injection of 0, 10, 20, 30, 40, 50, and 60 pA depolarizing currents for 400 ms. The spike number was determined to estimate the influence of rapamycin on the recorded neurons.

In all cases, biocytin (0.5%) was introduced into the intracellular solution to identify the morphological properties of the recorded neurons. After recording, the brain slices were immediately fixed in 4% paraformaldehyde in 0.1 M PB for 4 h at room temperature. Then, sections were rinsed with 3% hydrogen peroxide in 0.01 M PBS for 30 min. After thorough washing with PBS, the tissue was incubated with a goat anti-5-HT (1 : 500, Immunostar) antibody in PBS-NDS (pH 7.4) for 24 h, followed by incubation with Alexa 594 avidin D (1 : 1000, Invitrogen) and Alexa 488 donkey anti-goat (1 : 500, Invitrogen) antibodies in PBS for 6 h at room temperature. The sections were then observed, and images were captured with a confocal microscope (Olympus).

### 2.10. Statistical Analysis

Statistical data were calculated using GraphPad Prism 5 software. The results are expressed as the mean ± SEM. Two-way ANOVA with Bonferroni multiple comparisons tests or one-way ANOVA with Tukey's multiple comparisons post hoc tests were used for between-groups comparisons (e.g., the western blot data with surgery and drug administration as main effects). Student's paired *t*-test was used to analyze the differences between two groups (e.g., the difference in the numbers of p-mTOR-positive cells between the SNI-induced neuropathic pain group and the sham group). *P* values < 0.05 were considered significant.

## 3. Results

### 3.1. Spared Nerve Injury Produced Significant Mechanical Allodynia rather than Thermal Hyperalgesia in Rats

**Figure 1:**
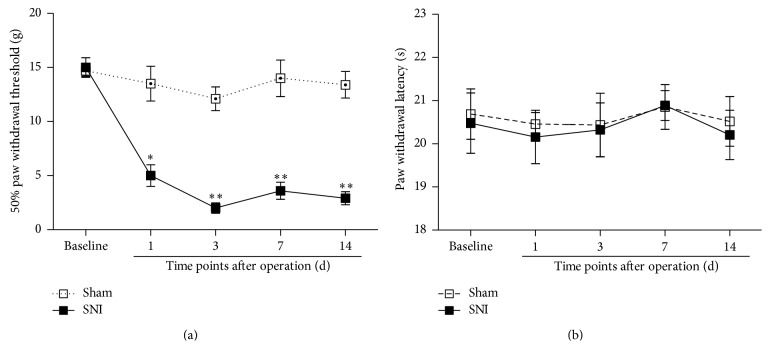
SNI produced significant mechanical allodynia but no thermal hyperalgesia in rats. (a) The 50% paw withdrawal threshold assessed by von Frey filaments was significantly lower after the SNI surgery (*n* = 8) compared to the sham (*n* = 8) group. Mechanical allodynia was obvious at day 1 and persisted for at least 2 weeks. (b) Paw withdrawal latency measured by radiant heat was not changed after the SNI surgery (*n* = 8) compared to the sham (*n* = 8) group (^*∗*^ *P* < 0.05 and ^*∗∗*^ *P* < 0.01 compared to the sham control at the same time point).

Spared nerve injury (SNI) produced increased nociceptive responses to innocuous mechanical stimulation (mechanical allodynia) of the ipsilateral hind paw in rats from as early as post-SNI operation day 1, and this effect was maintained for at least 2 weeks (Figure 1(a)). However, SNI had no impact on the latency of withdrawal to the radiant heat stimulus (no thermal hyperalgesia) (Figure 1(b)). In fact, our present data are consistent with a previous report demonstrating that the low mechanical threshold induced by SNI could persist even for 9 weeks after surgery, but there was no reported decrease in the hind paw withdrawal latency to the radiant heat stimulus [23].

### 3.2. p-mTOR Was Exclusively Expressed in Neurons within the RVM

**Figure 2:**
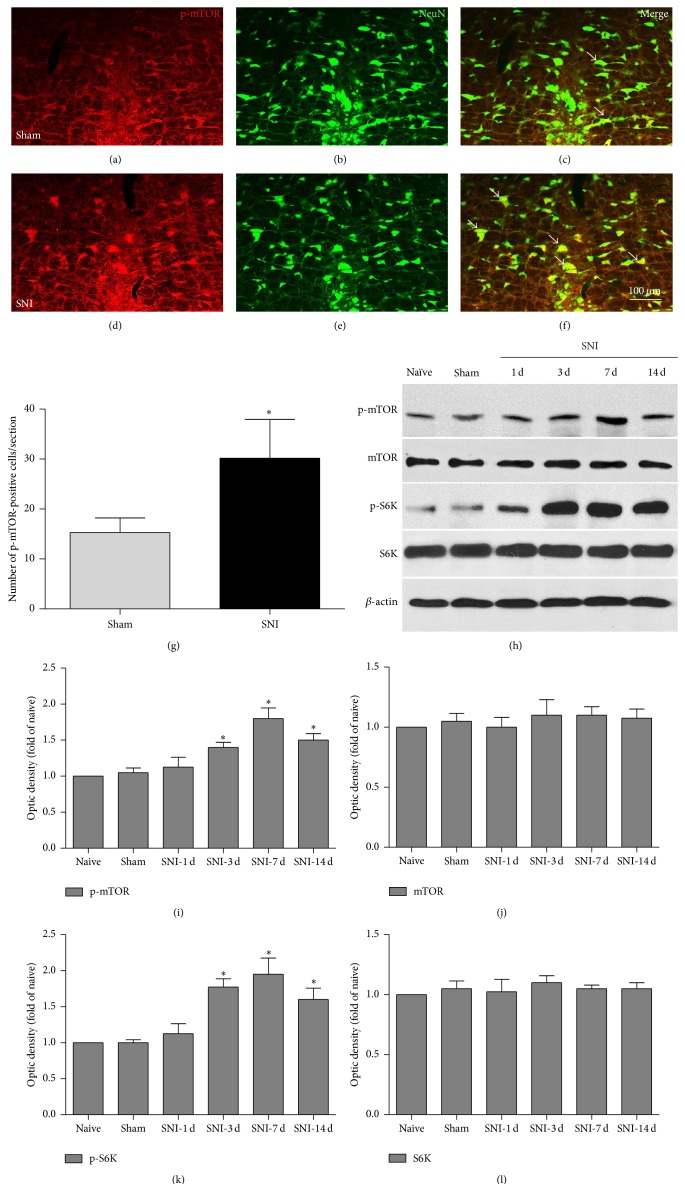
mTOR was remarkably activated in the RVM after SNI. (a)–(f) Double immunostaining showed that p-mTOR (red) was almost exclusively expressed in neurons (green). Single arrows indicate some typical double-labeled (yellow) cells. (a)–(g) Cell counting in the RVM shows that the number of p-mTOR-positive neurons was significantly increased 7 days after the SNI surgery (^*∗*^ *P* < 0.05 compared to the sham control group). Scale bars = 100 *μ*m. (h)–(l) The expression levels of total and phosphorylated mTOR (h, i, and j) and S6K (h, k, and l) were revealed by western blotting. Three days after SNI, phosphorylated mTOR and S6K (p-mTOR and p-S6K) in the RVM were significantly increased. Nonphosphorylated mTOR and S6K were not changed after SNI (^*∗*^ *P* < 0.05 compared to the naïve control group, *n* = 4).

By using double-immunofluorescence staining, we found that the activated form of mTOR, p-mTOR, was expressed in the RVM, and it was exclusively expressed by neurons based on the observation that p-mTOR was almost completely colocalized with NeuN, a marker for neurons, in rats in both the sham (Figures 2(a)–2(c)) and post-SNI day 7 groups (Figures 2(d)–2(f)).

### 3.3. The mTOR Signaling Pathway in the RVM Was Significantly Activated after SNI

We observed that the number of p-mTOR-positive neurons was significantly increased in the RVM on day 7 after SNI compared to the sham group (Figures 2(a), 2(d), and 2(f)), indicating that activation of mTOR in the RVM may contribute to SNI-induced neuropathic pain.

It has been reported that S6K, a main downstream substrate for mTOR, is involved in several intracellular processes, including neuronal plasticity and long-term memory [17]. Therefore, we used western blot analysis to further evaluate the mTOR signaling pathway, including mTOR and S6K, in the RVM after SNI. Compared with naïve control rats, the expression of both phosphorylated mTOR and phosphorylated S6K (p-mTOR and p-S6K) was not significantly altered in the sham rats (Figures 2(h), 2(i), and 2(k)). As indicated in Figure 2(h), p-mTOR and p-S6K were significantly elevated in the RVM 3 days after SNI, and phosphorylation was maintained for at least 14 days compared to the control group (Figures 2(h) and 2(i); p-mTOR: SNI D3: 1.42 ± 0.25; SNI D7: 1.86 ± 0.39; SNI D14: 1.49 ± 0.28-fold of naive control, ^*∗*^ *P* < 0.05; p-S6K: SNI D3: 1.75 ± 0.23; SNI D7: 1.96 ± 0.57; SNI D14: 1.61 ± 0.28-fold of naïve control, ^*∗*^ *P* < 0.05, *n* = 4). The change in p-S6K was similar to that in p-mTOR, indicating that the mTOR signaling pathway in the RVM was activated by SNI 3 days after surgery. Compared with the naïve control group, p-mTOR and p-S6K expression was slightly increased, but no significant difference was detected at 1 day after SNI (Figures 2(h), 2(i), and 2(k), p-mTOR: SNI D1: 1.17 ± 0.36-fold of naïve control, *P* > 0.05; p-S6K: SNI D1: 1.14 ± 0.38-fold of naïve control, *P* > 0.05, *n* = 4). Total mTOR and S6K were not changed in any of the groups in the present study (Figures 2(h), 2(j), and 2(l)). In summary, these data suggest that the mTOR signaling pathway, including mTOR and S6K, was activated in the RVM, which might indicate new protein synthesis and could result in changes in neuroplasticity.

### 3.4. Most Spinally Projecting Neurons in the RVM Expressed p-mTOR

**Figure 3:**
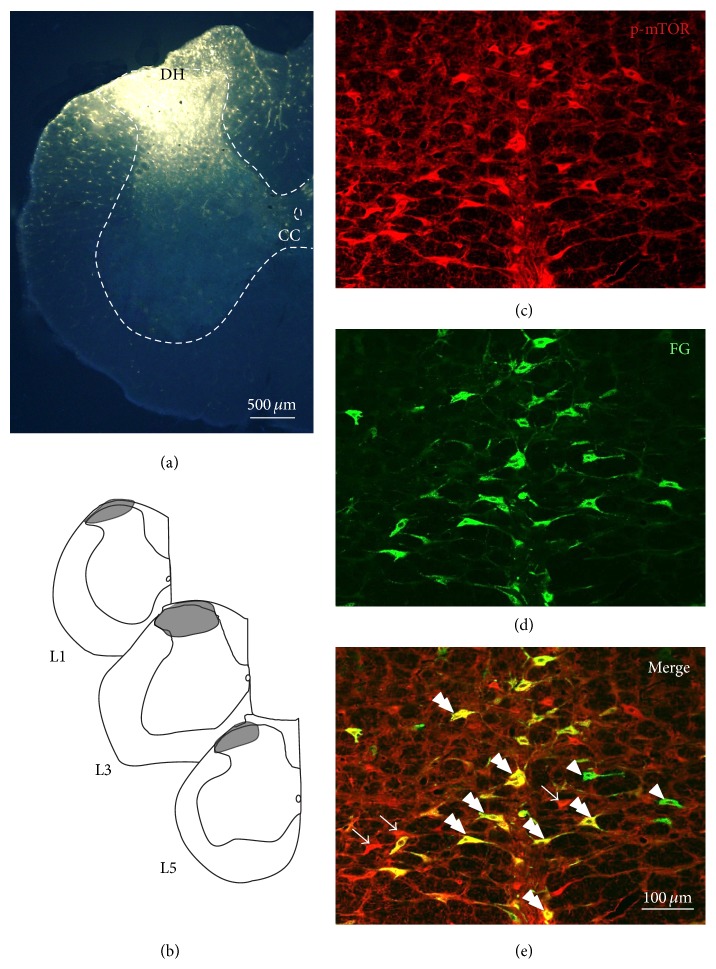
Most of the FG-labeled spinally projecting neurons in the RVM expressed p-mTOR. (a) Fluorescence photomicrograph showing the FG injection site in the lumbar spinal dorsal horn (SDH). (b) Camera lucida drawings show the rostrocaudal extent of the FG injection site at the different levels indicated, L1, L3, and L5. L1, L3, and L5 show the corresponding segments of the lumbar cord (DH: dorsal horn; CC: central canal). (c)–(e) Representative fluorescence photomicrographs showing p-mTOR (red) and FG (green) double-labeled neurons in the RVM. The double arrowheads indicate p-mTOR/FG double-labeled neurons (yellow), the arrowheads indicate FG single-labeled neurons, and the arrows indicate p-mTOR single-labeled neurons in the RVM. Scale bar = 500 *μ*m in (a) and 100 *μ*m in (e) (applied to (c)–(e)).

Injections of FG into the lumbar SDH (Figure 3(a) and 3(b)) resulted in many retrogradely labeled spinally projecting neurons in the RVM (Figure 3(d)). Although FG was injected unilaterally into the (left) lumbar SDH, 81.9% (203/248) of the retrogradely labeled spinally projecting neurons contained p-mTOR-immunoreactive (IR) staining (Figures 3(c)–3(e)), which indicates that more than three-quarters of the spinally projecting neurons in the RVM express p-mTOR.

### 3.5. The Upregulated p-mTOR Was Mainly Colocalized with 5-HTergic Neurons in the SNI Rats

**Figure 4:**
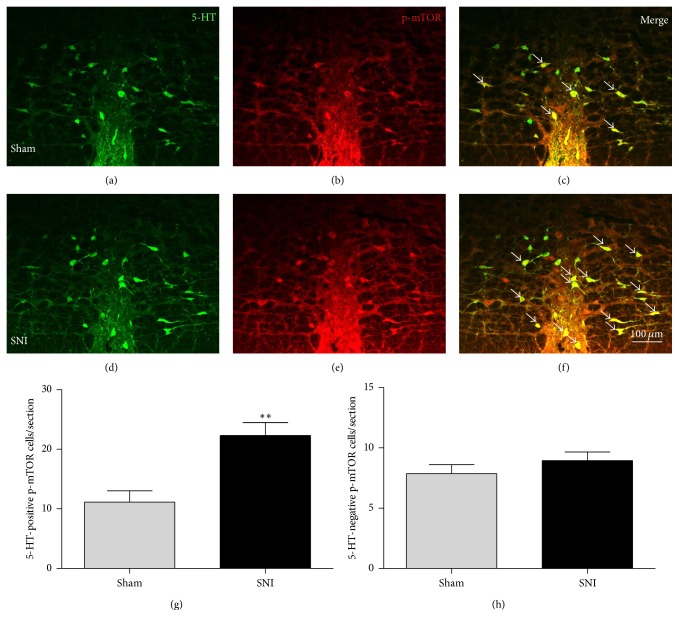
p-mTOR was mainly colocalized with 5-HTergic neurons in the RVM, and the number of 5-HT-positive p-mTOR neurons was significantly increased 7 days after SNI. (a)–(f) Representative fluorescence photomicrographs showing the expression of 5-HT (green) and p-mTOR (red) double-labeled neurons in the RVM in the sham control (a–c) versus the SNI 7-day group (d–f). Single arrows indicate p-mTOR/5-HT double-labeled (yellow) neurons. Scale bars = 100 *μ*m. (g) The average number of 5-HT-positive p-mTOR neurons per section increased significantly in the SNI rats 7 days after surgery compared to the sham (^*∗∗*^ *P* < 0.01). (h) In contrast, the average number of 5-HT-negative p-mTOR neurons did not change after SNI (*n* = 4 rats/group).

Because 5-HTergic neurons have previously been shown to be involved in the descending pain control pathway, particularly those localized within the RVM, we further investigated the relationship between 5-HT and p-mTOR. Our immunofluorescence staining results showed that a majority of the p-mTOR-IR neurons contained 5-HT (Figures 4(a)–4(f)). The number of 5-HTergic neurons was slightly increased within the RVM in the SNI rats, but no statistical significance was detected compared to the sham control group (25.52 ± 5.13 per section in the SNI D7 group* versus *20.8 ± 4.02 in the sham control group, *n* = 3 rats/group, *P* > 0.05). Interestingly, 5-HT-positive p-mTOR-IR neurons were remarkably increased in the RVM 7 days after SNI compared to sham rats (Figure 4(g)). In contrast, 5-HT-negative p-mTOR-IR neurons were not significantly altered 7 days after SNI (Figure 4(h)). These results indicate that SNI-induced neuropathic pain caused a substantial upregulation of p-mTOR, which primarily occurred in 5-HTergic neurons in the RVM.

### 3.6. Infusion of Rapamycin Rapidly Decreased the Upregulation of p-mTOR and p-S6K in the RVM in Brain Slices* In Vitro*

**Figure 5:**
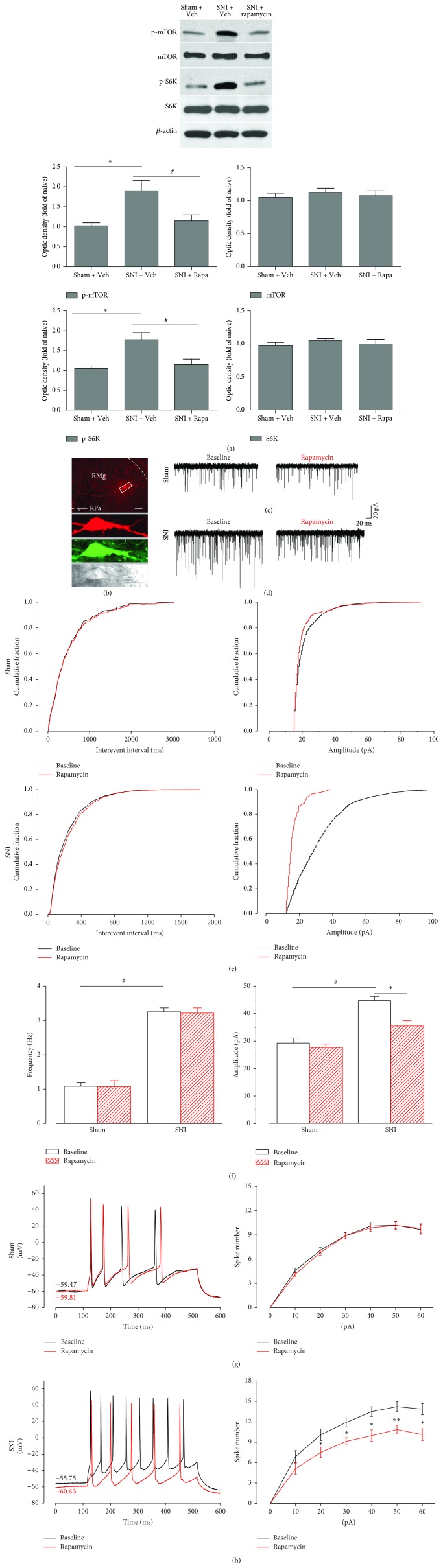
The neuronal excitability of 5-HTergic neurons in the RVM was significantly increased in SNI rats, which could be effectively impaired by rapamycin via a postsynaptic mechanism. (a)* In vitro* brain slice infusion revealed that rapamycin (250 *μ*M) rapidly inhibited the activation of mTOR in SNI rats. Consistently, in the absence of rapamycin, SNI produced a significant increase in the levels of phosphorylated mTOR and S6K (^*∗*^ *P* < 0.05 compared to the sham + vehicle group, *n* = 4). After treatment with rapamycin (250 *μ*M) on day 7, the upregulation of p-mTOR and p-S6K levels was remarkably decreased (^#^ *P* < 0.05 compared to the SNI + vehicle group, *n* = 4) in brain slices from SNI rats. (b) One representative whole-cell patched neuron in the RVM was injected with biocytin (labeled with Alexa 594, red). This cell also showed 5-HT immunoreactivity (Alexa 488, green). The same neuron is also pictured during whole-cell patching (RMg: raphe magnus nucleus; RPa: raphe pallidus nucleus). Scale bars = 100 *μ*m (above) and 25 *μ*m (below). (c)–(e) Superimposed samples and cumulative fraction results showing that rapamycin inhibited the amplitude rather than the frequency of spontaneous excitatory postsynaptic currents (sEPSCs) in the RVM 5-HTergic neurons. (c), (e) Bath application of rapamycin (250 *μ*M) had no effect on the frequency and amplitude of the sEPSCs in rats with sham surgery. (d), (e) Rapamycin (250 *μ*M) inhibited the frequency but not the amplitude of sEPSCs in rats with SNI. (f) Summarized results for the effects of rapamycin on sEPSCs in rats with SNI or sham surgery (^*∗*^SNI* versus* sham *P* < 0.05, *n* = 15; ^#^SNI + rapamycin* versus* sham + rapamycin, *P* < 0.05, *n* = 15). (g) Sample traces and average results showing that the number of action potentials (APs) in a train induced by the injection of step currents (400 ms, 0–60 pA) was not affected by rapamycin in the sham group (*n* = 15, *P* > 0.05, two-way repeated measures ANOVA). (h) Sample traces and average results showing that the number of APs in a train induced by the injection of step currents (400 ms, 0–60 pA) was significantly reduced by rapamycin in the SNI group (*n* = 15, *P* < 0.05, two-way repeated measures ANOVA). The Holm-Sidak post hoc test indicated that rapamycin decreased the spike number when currents of 20 (*t* = 2.50, ^*∗*^ *P* < 0.05), 30 (*t* = 2.62, ^*∗*^ *P* < 0.05), 40 (*t* = 2.34, ^*∗*^ *P* < 0.05), 50 (*t* = 3.34, ^*∗∗*^ *P* < 0.01), and 60 pA (*t* = 2.57, ^*∗*^ *P* < 0.05) were applied. It is worth noting that the resting membrane potential (RMP) was slightly hyperpolarized and that the amplitude of spikes was also slightly decreased in the SNI group.

As reported previously, rapamycin is a specific and effective inhibitor of mTOR. To investigate the inhibitory effect of rapamycin* in vitro*, we incubated brain slices containing the RVM with rapamycin (250 *μ*M) for 30 min. Thereafter, the RVM region was collected and assessed by western blotting. We found that SNI significantly increased the expression of both p-mTOR and its downstream substrate p-S6K (Figure 5(a), p-mTOR: SNI D7 + vehicle: 1.80 ± 0.48-fold of sham + vehicle, ^*∗*^ *P* < 0.05; p-S6K: SNI D7 + vehicle: 1.77 ± 0.45-fold of sham + vehicle, *n* = 4, ^*∗*^ *P* < 0.05), which is inconsistent with our tissue western blot data. Moreover, rapamycin (250 *μ*M) significantly reversed the upregulation of p-mTOR as well as p-S6K after 30 min of drug infusion (p-mTOR: SNI D7 + rapamycin: 1.11 ± 0.27-fold of naïve control* versus* SNI D7 + vehicle: 1.80 ± 0.48-fold of naïve control, *P* < 0.05; p-S6K: SNI 7d + rapamycin: 1.17 ± 0.29-fold of naïve control* versus* SNI D7 + vehicle 1.77 ± 0.45-fold of naïve control, *n* = 4, *P* < 0.05), indicating that rapamycin could rapidly inhibit the activation of mTOR after SNI* in vitro*.

### 3.7. The Excitability of 5-HTergic Neurons in the RVM Was Greatly Increased in SNI Rats, and It Could Be Effectively Impaired by Rapamycin via a Postsynaptic Mechanism

To further investigate the neuronal excitability of 5-HT and the effect of rapamycin on 5-HTergic neurons in the RVM, we carried out whole-cell patch-clamp recording. A total of sixty 5-HT-positive neurons (*n* = 60) from 12 rats were identified by biocytin introduction in combination with 5-HT immunofluorescence staining (Figure 5(b)). Moreover, the membrane characteristics of 5-HTergic neurons were quite different. They showed a relatively higher membrane capacitance (Cm) and a smaller membrane resistance (Rm) compared to other small neurons in the RVM, indicating that they have a larger membrane surface and smaller electrical resistance.

We first investigated the frequency and amplitude of spontaneous excitatory postsynaptic currents (sEPSCs) in the RVM neurons. Most of the 5-HTergic neurons recorded in the RVM that were responsive to rapamycin showed increased activity. Inconsistent with previous reports [29], the frequency and amplitude of sEPSCs were significantly increased after SNI (Figures 5(c)–5(f)), which indicates that the probability of presynaptic transmitter release and the postsynaptic neuronal excitability, respectively, was elevated. Subsequently, we used rapamycin (250 *μ*M) to evaluate whether the enhanced presynaptic and postsynaptic excitability could be reversed. We found that the amplitude (baseline, 45.26 ± 1.89 pA; rapamycin, 35.83 ± 2.58 pA. *P* < 0.05, *n* = 15, paired *t*-test), but not the frequency (baseline, 3.41 ± 0.12 Hz; rapamycin, 3.29 ± 0.33, *P* > 0.05, *n* = 15, paired *t*-test), of the sEPSCs was inhibited by rapamycin application in rats with SNI but not in the sham control rats (frequency: baseline, 1.22 ± 0.11 Hz; rapamycin, 1.20 ± 0.19 Hz. *P* > 0.05, *n* = 15, paired *t*-test; amplitude: baseline, 29.79 ± 1.80 pA; rapamycin, 28.14 ± 1.28 pA. *P* > 0.05, *n* = 15, paired *t*-test) (Figures 5(c)–5(f)). These results suggest that rapamycin can decrease the postsynaptic excitability of 5-HTergic neurons in the RVM after SNI.

We next compared the effects of rapamycin on the action potentials of the 5-HTergic neurons and determined that the spike number of the recorded neurons was obviously increased after SNI (Figures 5(g) and 5(h)). Rapamycin (250 *μ*M) did not change the spike number in recorded neurons from the sham group (*F*_(1,62)_ = 2.25, *P* > 0.05, *n* = 15, two-way repeated measures ANOVA) (Figure 5(g)). However, in the presence of rapamycin, the spike number of 5-HTergic neurons in the SNI group was significantly reduced (*F*_(1,48)_ = 8.56, *P* < 0.05, *n* = 15, two-way repeated measures ANOVA) (Figure 5(h)). These results indicate that rapamycin inhibited the excitability of 5-HTergic neurons in the RVM under neuropathic pain conditions.

### 3.8. Microinjection of Rapamycin into the RVM Potently Alleviated Mechanical Allodynia during the Maintenance but Not the Induction of SNI-Induced Neuropathic Pain

We preliminarily investigated nociceptive behaviors in the SNI rats mentioned above. In agreement with previous reports [22], significant mechanical allodynia rather than thermal hyperalgesia was observed in the present study (Figure 1). Based on this observation, we next used mechanical PWT instead of heat PWL to assess the nociceptive behaviors.

**Figure 6:**
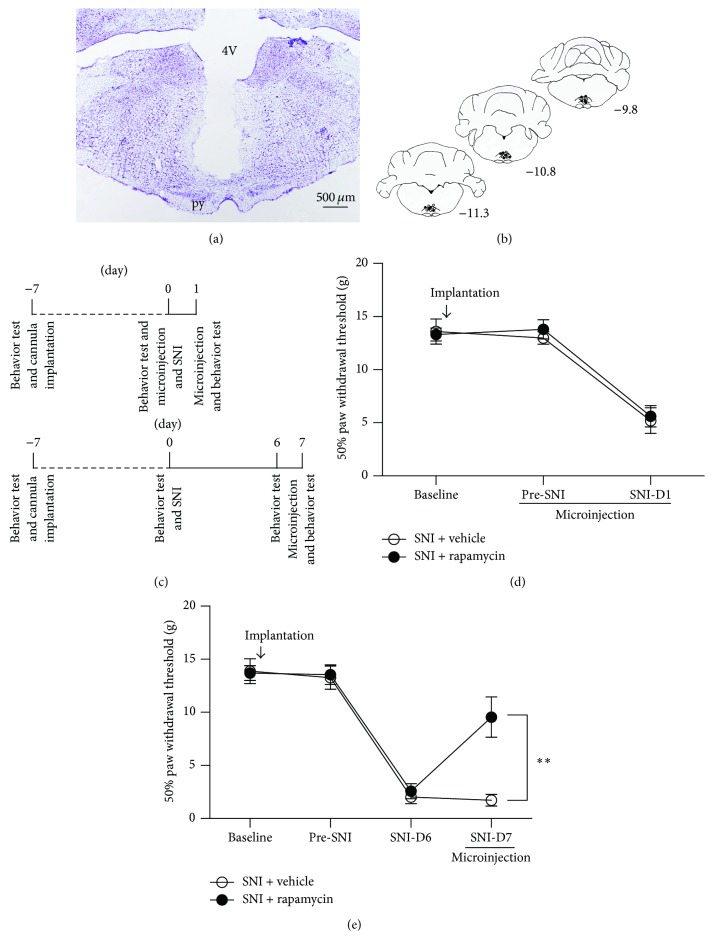
Intra-RVM microinjection of the mTOR inhibitor rapamycin (250 *μ*M) partially reversed established mechanical allodynia (maintenance stage) but showed no effect on the induction stage of SNI-induced neuropathic pain in rats. (a) Representative Nissl-stained section showing injection sites within the RVM (4V: 4th ventricle; py: pyramidal tract). Scale bar = 500 *μ*m. (b) Camera lucida drawings showing the cannula tip placements in rats injected with rapamycin (black circles, *n* = 14) or vehicle (white circles, *n* = 14) in the RVM. The numbers correspond to the distance in millimeters (mm) posterior to bregma in the brain. (c) Experimental schedule. The behavioral tests, cannula implantation, microinjection, and SNI surgery were performed as indicated in the schedule. (d) Intra-RVM microinjection of rapamycin (250 *μ*M) before SNI (Pre-SNI) and 1 day after SNI (SNI-D1) could not alleviate mechanical allodynia 30 min after the SNI-D1 injection compared to vehicle injection (*n* = 7 rats/group). (e) Microinjection of rapamycin (250 *μ*M) into the RVM 30 min before the behavioral test significantly reduced mechanical allodynia at 7 days after SNI (SNI-D7) compared to vehicle injection (^*∗∗*^ *P* < 0.01, *n* = 7 rats/group).

To determine whether rapamycin could prevent the development of the induction stage of mechanical allodynia, we microinjected rapamycin via a cannula implanted into the RVM (Figures 6(a) and 6(b)) immediately before SNI surgery and at day 1 after SNI, which was followed by behavioral testing 30 min later (Figure 6(c)). After treatment with rapamycin, the PWT in the SNI + rapamycin group showed no significant change compared to that in the SNI + vehicle control group at day 1 after SNI (Figure 6(d)), indicating that rapamycin could not alleviate the neuropathic pain induced by SNI in the induction stage. Subsequently, we investigated whether rapamycin could reverse established neuropathic pain. On day 6 after SNI, the rats demonstrated typical increased nociceptive responses to nonnoxious mechanical stimulation (Figure 6(e)). Compared with the vehicle control group, the mechanical allodynia was significantly reduced after microinjection of rapamycin into the RVM on day 7 after SNI (Figure 6(e)). Because our biochemical results showed that the amount of both p-mTOR and p-S6K was increased after SNI (Figures 2(h), 2(i), and 2(k)) and because rapamycin infusion into the brain slices could effectively reverse the upregulated level of p-mTOR and p-S6K (Figure 5(a)), we concluded that the effect of rapamycin in partially reversing the mechanical allodynia was exerted via inactivation of the mTOR signaling pathway, thus decreasing the excitability of 5-HTergic spinally projecting neurons in the RVM.

## 4. Discussion

In the current study, we provide the first demonstration of the following: (1) mTOR is expressed in the RVM region and can be activated in nerve injury-induced neuropathic pain; (2) this mTOR is largely expressed in 5-HTergic neurons, which mainly comprise the descending pain control pathway; (3) inhibition of the activated mTOR restores the overexcitability of the 5-HTergic neurons to normal; and (4) inactivation of mTOR by intra-RVM rapamycin microinjection alleviates established hyperalgesia (abolished at the maintenance stage of neuropathic pain) rather than influencing the beginning priming (induction stage) of neuropathic pain. These findings suggest that the mTOR signaling pathway in the RVM is involved in the maintenance of nerve injury-induced neuropathic pain and that inhibition of mTOR in the RVM could effectively alleviate neuropathic pain in SNI rats.

Due to the importance of the descending pain control pathway in mammals [2], many studies have been performed to elucidate the mechanisms underlying this critical pathway. It has been demonstrated that blockade of RVM activity with lidocaine produced conditioned place preference (CPP), which is linked to pain relief, in nerve injury models, indicating that descending pain facilitation pathways modulate injury-induced spontaneous tonic pain [30]. Wei et al. [3] have reported that selectively depleting functional 5-HT phenotypes in RVM neurons with shRNA interference (RNAi) of tryptophan hydroxylase-2 (Tph-2, the rate-limiting enzyme in the synthesis of neuronal 5-HT) attenuated tissue or nerve injury-induced allodynia and hyperalgesia. This finding provides strong evidence that descending 5-HT from the RVM is an important contributor to pain facilitation during the development of persistent pain. Recently, by taking advantage of optogenetic methods, optogenetic stimulation in Tph2-channelrhodopsin 2 (ChR2) transgenic mice was shown to decrease both mechanical and thermal pain thresholds [31]. However, in contrast, several studies showed the opposite results [32–35], that 5-HT from the RVM is important for the descending inhibitory pathway. These controversial arguments regarding whether RVM 5-HT plays a facilitatory or inhibitory role might be explained by the different subtypes of 5-HT receptors located in the SDH, according to the reports. For example, 5-HT_3_ receptors are reported to mediate descending facilitation and to contribute to pain hypersensitivity [36], whereas the activation of 5-HT_2_ receptors can potentiate glycine release in the SDH to inhibit pain transmission [35]. In addition, a previous study also provided evidence that, under conditions of experimental pain, activation of 5-HT_7_ receptors leads to antinociceptive effects in the spinal cord [37]. In the present study, we found that 5-HTergic neurons were slightly, although not significantly, increased after nerve injury. However, the excitability of these 5-HTergic neurons was elevated after SNI (Figure 5). Rapamycin could effectively inhibit 5-HT overexcitability and thus attenuate hyperalgesia (Figure 6), which indicates that 5-HT in the RVM is probably involved in the descending pain facilitation pathway under nerve injury-induced neuropathic pain conditions.

mTOR has been extensively studied in tumors [38], cardiovascular diseases [39], and neurodegenerative disorders [40, 41]. Recently, emerging evidence has indicated that mTOR plays a role in pain processing, and it is becoming clear that mTOR is important in the regulation of nociception, at both the peripheral and spinal cord levels [10–17]. To date, however, no report has investigated mTOR at the supraspinal level and its role in nociceptive modulation. Here, we provide potent evidence that mTOR contributes to neuropathic pain by increasing the neuronal excitability of 5-HTergic neurons in the RVM, thus potentiating descending pain facilitation.

mTOR regulates protein translation through multiple factors. 4E-BP1/2 and S6K are involved in the regulation of cell physiology through the modulation of protein synthesis [42]. 4E-BP1/2 inhibits the interaction of the cap-binding translation initiation factor eIF4E with other elongation factors, which is a key regulatory process in translation. mTOR-mediated phosphorylation of 4E-BP1/2 releases this inhibition, allowing translation initiation to proceed. S6K-mediated phosphorylation of S6 promotes the unwinding and initiation of translation of a subgroup of mRNAs called 5′-terminal oligopyrimidine tract (TOP) mRNAs. TOP mRNAs encode ribosomal proteins and elongation factors 1a and 2, which are important in translational control [43]. In the present study, we detected that p-mTOR and p-S6K levels were significantly elevated in the RVM after SNI (Figure 2), which suggests that mTOR-mediated protein translation and synthesis are increased. The upregulated p-mTOR was mainly coexpressed with 5-HT (Figure 4). In contrast, the number of 5-HTergic neurons was slightly increased within the RVM in the SNI rats, but no statistical significance was detected compared to the sham control group. Therefore, the synthesis of 5-HT in the RVM may not obviously increase. However, by using whole-cell patch recording, we found that the 5-HTergic neurons were overexcited, with significant increases in the amplitude and frequency of sEPSCs as well as the number of action potentials (Figure 5). In addition, rapamycin inhibited only the amplitude and not the frequency of sEPSCs (Figure 5); we thus propose that the postsynaptic overexcitability of the 5-HTergic neurons, which primarily depends on an increase in glutamate receptors, is mainly due to the activation of mTOR. It has been reported that mTOR signaling can potentiate the insertion of AMPA (*α*-amino-3-hydroxy-5-methyl-4-isoxazole-propionic acid) receptors into the postsynaptic membrane and lead to long-term potentiation (LTP) [44, 45]. Thus, the data collected from our immunofluorescence staining and electrophysiology are consistent with previous reports and suggest that the activation of mTOR might lead to an increase in AMPA receptors and their insertion into the postsynaptic membrane, resulting in the elevated neuronal excitability of the 5-HTergic neurons in the RVM.

As an effective immunosuppressant, rapamycin is widely used to prevent transplant rejection. Chronic treatment of patients with mTOR inhibitors is associated with an increased incidence of pain [46, 47], including the possible development of complex regional pain syndrome (CRPS) [48, 49]. Other animal studies have also reported similar results [50, 51]. These conflicting results might be due to the following reasons: (1) the drug concentration of rapamycin or its analogues was not the same as in the other reports in which the inhibition of mTOR produces antinociception; (2) long-term treatment might lead to feedback activation of other pronociceptive signal proteins or molecules; and (3) intrathecal administration of rapamycin (at the spinal cord level) might play a very complicated role in pain transmission with unknown mechanisms.

The present study used intra-RVM, instead of intrathecal, administration of rapamycin (250 *μ*M, 30 min before the behavioral tests), and this treatment remarkably attenuated the nociceptive behaviors induced by SNI (Figure 6). Moreover, intra-RVM rapamycin treatment was effective on day 7 after SNI (the maintenance stage of neuropathic pain) but not on day 1 after SNI (the induction stage). The behavioral pharmacological data suggest that inhibition of mTOR in the RVM at the late phase (maintenance stage) of neuropathic pain might be effective, even though pain has already been well established. By contrast, inhibition of mTOR in the RVM had no effect on the development (induction stage) of neuropathic pain. All of these results are consistent with our biochemical data showing that RVM p-mTOR was not greatly enhanced at day 1 but showed a significant increase at 7 days after SNI (Figure 2).

Combining our current results with previous findings, we conclude that the specific inhibition of mTOR by rapamycin in the RVM is a promising avenue for the management of neuropathic pain. This effect probably occurs via deactivation of 5-HTergic spinally projecting neurons in the RVM, which are required for descending pain facilitation. Nonetheless, the present study also has some limitations. Due to the lack of a specific mTOR activator, reverse experiments involving the activation of mTOR in the RVM, which should produce or enhance nociception, are difficult to achieve. Moreover, optogenetic methods as well as transgenic animals should be further introduced to confirm the role of mTOR in the RVM, not only in neuropathic pain but also in inflammatory pain.

## 5. Conclusion

Through the deactivation of 5-HTergic spinally projecting neurons in the RVM and thus the weakening of descending pain facilitation, specific targeting of the activation of mTOR in the RVM is a promising avenue for the management of neuropathic pain.

## Figures and Tables

**Table 1:** Antisera used in each group.

Group	Primary antisera	Secondary antisera
p-mTOR/NeuN	Rabbit anti-p-mTOR (1 : 200, Cell Signaling Technology, Danvers, MA, USA) Mouse anti-NeuN (1 : 500, Millipore, Temecula, CA, USA)	Alexa 594 donkey anti-rabbit (1 : 500, Invitrogen, Camarillo, CA, USA) Alexa 488 donkey anti-mouse (1 : 500, Invitrogen)

p-mTOR/FG	Rabbit anti-p-mTOR (1 : 200, Cell Signaling Technology) Guinea pig anti-FG (1 : 200, Protos Biotech, New York, NY, USA)	Alexa 594 donkey anti-rabbit (1 : 500, Invitrogen) Alexa 488 donkey anti-guinea pig (1 : 500, Invitrogen)

p-mTOR/5-HT	Rabbit anti-p-mTOR (1 : 200, Cell Signaling Technology) Goat anti-5-HT (1 : 500, Immunostar, Hudson, WI, USA)	Alexa 594 donkey anti-rabbit (1 : 500, Invitrogen) Alexa 488 donkey anti-goat (1 : 500, Invitrogen)
